# Acute flaccid myelitis: not uncommon in rural Uganda?

**DOI:** 10.1093/braincomms/fcad246

**Published:** 2023-10-18

**Authors:** Sam Olum, Charlotte Scolding, Venice Omona, Kansiime Jackson, Neil Scolding

**Affiliations:** Department of Internal Medicine, Gulu Medical School, Gulu University, Gulu, Uganda; St Mary’s Hospital, Lacor, Gulu, Uganda; Department of Internal Medicine, Gulu Medical School, Gulu University, Gulu, Uganda; St Mary’s Hospital, Lacor, Gulu, Uganda; THS, Faculty of Medicine, University of Bristol, Bristol, BS10 5NB, UK; Royal United Hospital, Bath, BA1 3NG, UK; Department of Internal Medicine, Gulu Medical School, Gulu University, Gulu, Uganda; St Mary’s Hospital, Lacor, Gulu, Uganda; Department of Internal Medicine, Gulu Medical School, Gulu University, Gulu, Uganda; St Mary’s Hospital, Lacor, Gulu, Uganda; Department of Internal Medicine, Gulu Medical School, Gulu University, Gulu, Uganda; St Mary’s Hospital, Lacor, Gulu, Uganda; Royal United Hospital, Bath, BA1 3NG, UK; Gloucestershire Hospitals NHS Trust, Cheltenham, GL53 7AN, UK

**Keywords:** acute flaccid myelitis, poliomyelitis, Uganda, sub-Saharan Africa

## Abstract

Acute Flaccid Myelitis is a paralytic illness with significant similarities to poliomyelitis, and which affects predominantly children. It was first fully delineated only in 2014 in the USA, occurring in epidemic clusters with a likely overall increasing incidence. It has subsequently rapidly been identified in Europe, the UK, and Australasia and the Far East, confirming it to be an emerging, global, infectious neurological disease. It has, however, been very little studied in low- and middle-income countries—reflecting partly of the global imbalance in science and medical research, and partly the extremely low provision of neurological care in most low- and middle-income countries: Uganda currently has no specialized neurology services outside the capital Kampala. During extended visits over a 2-year period with involvement in acute adult and paediatric internal medicine, one of us (NS) encountered at least six new patients with acute flaccid myelitis, suggesting that both the geographical reach and the frequency of the disorder may be significantly greater than previously thought. Here, these cases are described together with their clinical features and, where available, course and (limited) investigation results. These observations have significant implications concerning the current, and potentially the future geographical spread of the disease, and its clinical phenomenology. In addition, they highlight serious problems concerning the global applicability of the current Acute Flaccid Myelitis diagnostic criteria.

## Teaser

Acute Flaccid Myelitis is a paralytic, (mainly) childhood illness resembling poliomyelitis, first delineated in 2014 in the USA, and now identified across the global north. Scolding *et al.* describe six Acute Flaccid Myelitis individuals in northern Uganda, the first report in Sub-Saharan Africa. They highlight the inapplicability of current diagnostic criteria in low-income settings.

## Graphical Abstract

**Graphical abstract:**
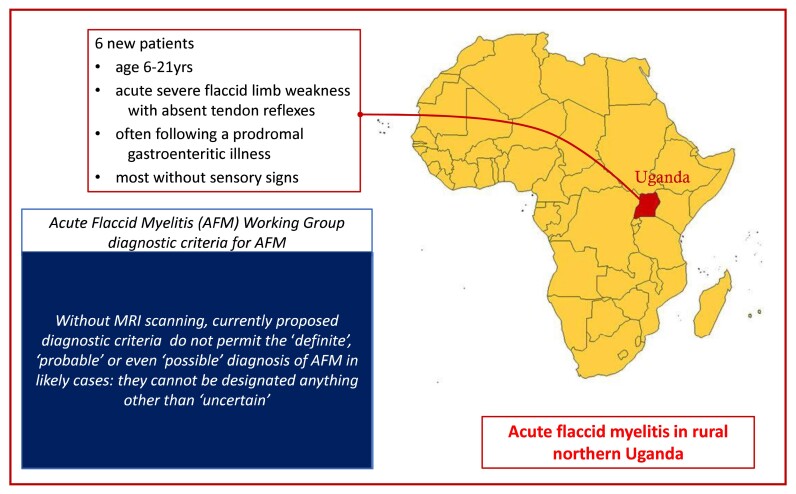

## Introduction

Acute Flaccid Myelitis (AFM) is a paralytic illness with significant similarities to poliomyelitis, and which predominantly affects children. It is an emerging neurological illness,^1^ first described and fully delineated only in 2014 in the USA,^2-4^ rapidly then identified in Europe,^5,6^ the UK^7,8^ and Australasia.^9^ In the USA it occurs in epidemic clusters^2,4^ but has a likely overall increasing incidence;^1,7^ its propensity to cause larger or even global epidemics remains unexplored.

There are reports of cases in southern India, South Africa and South America,^10-13^ and suggestions that it is a global disease,^1,3^ but in truth it has been very little studied in low and middle income countries (LMICs). This may be partly a reflection of the global imbalance in science and medical research, and partly of the extremely low provision of neurological care in most LMICs.^14,15^

Uganda currently has no specialized neurology services outside the capital Kampala (and no postgraduate neurology training programmes: in fact, there are none in East Africa^16^). During extended visits over a 2-year period with involvement in acute adult and paediatric internal medicine, one of us (NS) encountered at least six cases highly likely to represent AFM, suggesting that both the geographical reach and the frequency of the disorder may be significantly greater than previously thought. These observations have significant implications concerning the current, and potentially the future geographical spread of the disease, and also for the global applicability of the current diagnostic criteria.

### Ethics

Ethics permission was granted by Lacor Hospital Institutional REC (LACOR-2022-120).

### Case descriptions

#### Case 1

A previously well 8-year-old girl presented with an inability to walk, sit, or hold anything with either hand. Three days earlier she had had several hours’ of vomiting but described no headache, and no diarrhoea. Her vomiting settled, but within hours she developed limb weakness—she ‘spilled water from jerrycan coming back to her home.’ She went to bed crying with lower limb pain; the next day she could not sit, walk or hold anything; she was incontinent of urine. On examination, she was reported to be alert, but with flaccid weakness of all four limbs; there were no cardiovascular, respiratory or abdominal findings and no rash.

She was later transferred to our hospital, where her mother indicated that her legs were spontaneously improving. Re-examination (now ∼1 month from onset) showed normal cranial nerves but with slight but definite neck stiffness. Both upper limbs were plegic from the shoulders down. All her arm tendon reflexes were absent and tone was reduced. In her lower limbs, tone was normal, and power varied between Medical Research Council (MRC) Grades III–IV, broadly symmetrically. Her leg tendon reflexes were all present and symmetrical, and her plantar responses were mute. Sensation was normal throughout.

Limited investigations were undertaken: Full blood count was normal, no malarial parasites were seen. Her CSF was clear and colourless, with <5.0 wbc/mm^3^; Pandy test, CrAg and India Ink testing were all negative.

Her mother removed her from hospital after just a few days; her overall outcome was therefore unknown.

#### Case 2

A 7-year-old previously well boy presented with a 5-day history of sore throat and ‘high fevers’, followed 48 hours later by progressive weakness of the arms and legs; he was now unable to walk. He had no vomiting or diarrhoea, no rash, and there were no visual, speech, swallowing or sphincter symptoms.

On examination he was afebrile. There was no neck stiffness and the cranial nerves were normal. All four limbs were hypotonic with asymmetrical MRC Grade 2–3 weakness throughout (left weaker than right). All his tendon reflexes were absent, and the plantar responses were mute. Sensation throughout was normal.

Full blood count was normal and blood film revealed no malarial parasites.

With a putative diagnosis of AFM, he was treated with dexamethasone 12 mg/d. Two days later there was some improvement, but he later became breathless, with increased neck and shoulder weakness. He was transferred for monitoring to the intensive care unit but did not require ventilation. His breathing improved again after a few days, and he was transferred to an open ward, but then was taken home by his family whilst still showing significant weakness. No follow-up examination was possible.

#### Case 3

A 9-year-old girl was admitted with a story of headache followed by rapidly progressive weakness of all four limbs, though worse on the right, leaving her unable to stand unaided. She had previously been well with the significant exception of an identical episode of weakness of all four limbs some 18 months previously with near-complete spontaneous recovery.

On examination, the eyes and cranial nerves were normal, but she was tetraparetic, with arm weakness more prominent on the right and a paraplegia. The tendon reflexes in the right arm were brisk; all others were sluggish. Sensation was normal throughout. Her spinal fluid was normal, as was a full blood count. With a presumptive diagnosis of AFM, she was treated with oral dexamethasone.

#### Case 4

A 6-year-old boy presented a month after the onset of a mild headache followed by rapidly progressive weakness of all four limbs. The legs were more affected than the arms; he was unable to walk at presentation. Bladder function was not affected. He had had an identical episode some 8 months previously with complete spontaneous recovery. He had otherwise previously been well.

On examination, the cranial nerves were normal and there was no meningism. He had a flaccid tetraparesis. The upper limbs were MRC Grade III–IV power proximally, Grade IV distally. The lower limbs were Grade I–II proximally, Grade II distally. Tone was reduced in all four limbs and he was areflexic. Sensation was normal throughout.

The clinical impression was one of a recurrence of AFM.

#### Case 5

A 21-year-old female presented 3 weeks after the onset of diarrhoea, lasting 3 days, together with low back pain, followed a few days later by rapid onset of weakness in her right leg, then 3 days later of her left leg leaving her unable to walk or stand. Her weakness was combined with complete loss of sensation from the onset, and also of bladder and bowel function. There were no symptoms above the waist, and she had previously been well.

**Figure 1:**
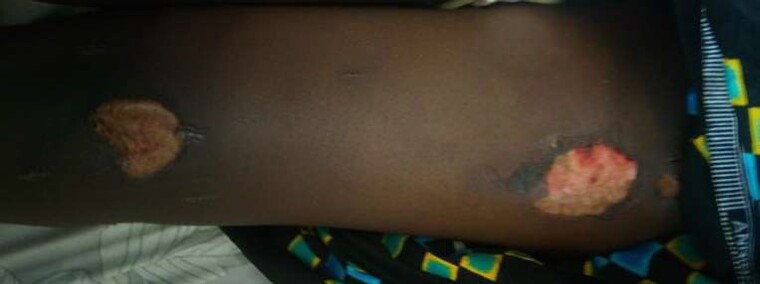
Painless burns, and scars from traditional medicine in the leg, in a child with AFM.

On examination, she was alert and articulate, afebrile and had no neck stiffness. The cranial nerves and upper limbs were normal. Both legs were completely plegic, hypotonic and with absent tendon jerks. Her plantar responses were absent. She had total absence of all sensory modalities below the waist, with three painless burns in her left upper leg (Fig. 1). There were also linear scars on her legs following a traditional medicine attempt to cure her symptoms (also see Fig. 1).

The clinical impression was of acute myelitis, currently flaccid (3 weeks from onset).

Her Full blood count was normal, and her CSF was likewise normal, with no cells and a normal total protein level. CrAg testing was negative.

By 10 days later there had been some return of pain sensation proximally in both legs, but no return of power; both legs remained deeply flaccid, and the diagnosis of AFM seemed clinically likely.

#### Case 6

A 15-year-old girl developed diarrhoea 11 days before admission, accompanied by headache but no vomiting, followed by ascending weakness of both legs rendering her unable to walk. She then developed bilateral arm weakness, together with difficulty micturating. There was no back (or other) pain, no sensory symptoms, and no speech, swallowing or visual symptoms.

On examination, her cranial nerves were normal but she had mild neck stiffness. She had flaccid weakness of the arms and legs, the left weaker than the right, the lower limb extensors affected more than the flexors and the upper limb flexors more than the extensors. All her tendon reflexes were absent, her plantar responses were also absent. There were no sensory abnormalities.

The pure motor picture, bladder involvement and neck stiffness all rather mitigated against Guillain Barre syndrome, and the clinical impression was of AFM.

## Discussion

The diagnosis of AFM in each of these six cases was almost entirely a clinical one, made on the balance of probabilities and with the support of limited investigations—no spinal cord imaging, limited CSF analysis, no clinical neurophysiology, little testing for specific pathogens. The principal alternative diagnoses, however, in each case seem very unlikely. Guillain Barre syndrome was often considered, perhaps especially in Case 6 which had an ascending character to the progressive weakness, but the absence in this case (and 4/5 of the other cases) of any sensory involvement, and (in every case) of cranial nerve involvement militated against an inflammatory neuropathy. To a perhaps lesser extent, the extremely rapid progression, significant asymmetry (in most) of limb weakness, and normal CSF Pandy curve would also have been atypical for Guillain Barre syndrome. Acute transverse myelitis was also considered, especially in Case 5, the only case with (prominent) sensory impairment, and in whom also there was a paraplegia rather than a tetraparesis. It remains possible that transverse myelitis was the explanation, but the persistence of complete areflexia and flaccidity in the legs even after 5–6 weeks militates strongly against this. Spinal cord MRI scanning, had it been available, would very likely have resolved the question.

Assuming our cases can indeed be accepted as AFM, some observations may be offered. These six cases were seen during the course of three 6-week spells in a single hospital in northern Uganda. They were encountered serendipitously: no relevant AFM research project was underway, nor any specific surveillance programme—and so, while speculative, it is highly likely that there were further incident cases during these same periods. The absence of any published case reports of AFM from Uganda (or indeed across sub-Saharan Africa) must be partly explained by the extremely low provision of neurological specialist care in many if not most LMICs.^14,15^ Uganda currently has no specialist neurological services outside Kampala (and very few MRI scanners), and there are no postgraduate neurology training programmes in East Africa:^16^ currently, those physicians interested in acquiring neurological specialist training leave Uganda to acquire it, principally at present through relationships with US partners. Therefore, the endemic incidence of AFM in sub-Saharan Africa may be substantially higher than that seen in the global North—and as a likely viral illness this has significant ramifications.

A number of viruses known to be associated with paralysis are comparatively common in East Africa, including West Nile virus, Japanese B Encephalitis virus, dengue, zika and chikungunya, offering one potential explanation for a higher incidence of flaccid myelitis. The absence of other clinical features of any of these viruses, however, suggests this is unlikely, and may imply that the same viruses implicated in AFM in the global north—principally, enterovirus D68, although there is evidence from the USA that the virus responsible differs between peak and nonpeak years^4^—are the more likely culprits. (Polio virus itself is not a plausible culprit for the currently reported cases: the WHO surveillance programme in Uganda tests samples from all cases nationally of acute (non-traumatic) flaccid weakness for poliovirus; none has been identified.^17^) Both the causal virus(es) and the true incidence of AFM in East Africa are pressing questions requiring prompt research.

Firm conclusions cannot be drawn from two of the more unusual clinical aspects of these cases—first, the striking sensory findings in Case 5, and second, the history of identical previous episodes in two of the six cases. Sensory symptoms are reportedly common in AFM, but sensory loss sufficient to allow painless burns is not. Again, the lack of MRI scanning ability must lend caution to interpreting this finding.

Similarly, we have been unable to identify any published examples of relapse in AFM; it is only recorded as a monophasic illness.^1^ Whilst not impossible to imagine, it should be re-emphasized that neither individual was seen by any of the authors during his or her first episode of weakness, nor were any records available, and so the possibility of some alternative cause of the first episode cannot be excluded. Still, both findings merit recording and pursuing in more definitive prospective studies.

**Figure 2:**
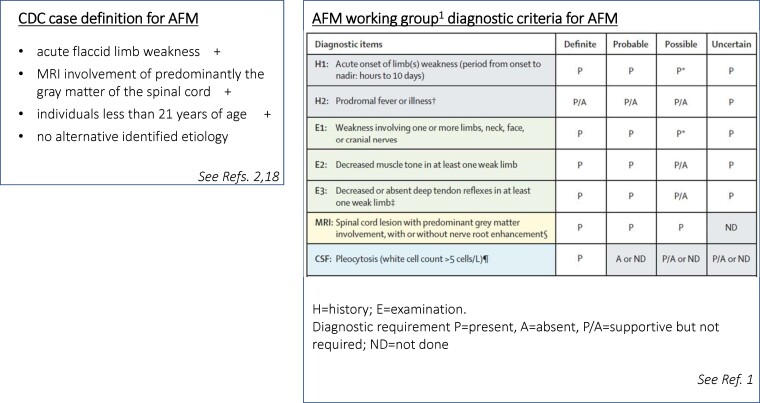
Currently proposed diagnostic criteria and case definition for AFM.

This lack of availability of more sophisticated investigations highlights an important aspect of the current diagnostic approach recommended in the international literature concerning AFM. According to the proposed diagnostic guidelines^1^ (Fig. 2), the absence of MRI scanning prevents any of our cases from being considered ‘definite’. More seriously problematic is that, without MRI scanning, these guidelines also exclude both a ‘probable’ and even ‘possible’ diagnosis for the currently presented cases: none could be designated anything other than ‘uncertain’. Similarly, the (separate) centers for disease control and prevention case definition does not permit the diagnosis without MRI scanning—‘individuals <21 years of age with acute flaccid limb weakness AND MRI involvement of predominantly the grey matter of the spinal cord without identified aetiology.’^2,18^ These criteria are therefore anything but globally inclusive, and we propose that they need urgent revision, for two reasons.

First, excluding the vast majority of low-income countries’ cases cannot be acceptable. Sub-Saharan Africa accommodates 15% of the world’s population and carries 25% of the world’s disease burden^19^—but produces just 1.3% of global science publications^20^ (and the great majority of these are from a handful of institutions in a single country, South Africa). Revising highly exclusive approaches to disease diagnosis would be one small step towards addressing this major global imbalance.

More specifically but no less importantly, our observations suggest AFM occurs at a significant frequency in East Africa. Accurate data concerning the frequency of the disorder, improving its recognition, and improved knowledge of outcomes, has significant implications for health policy or practice, not only locally but internationally, given the apparently increasing prevalence of the disorder in high-income countries, regardless of whether this is related to any potentially higher endemic incidence in LMICs. Revising the diagnostic criteria to make them truly globally applicable would significantly improve our ability to study this serious disorder.

One possible solution we propose is to increase the weight given to just two simple clinical criteria—the absence of objective sensory involvement, and the absence (after, say 4–6 weeks) of spasticity or hyper-reflexia. If these two criteria are met, the diagnosis should be categorized as ‘probable’, not ‘uncertain’. The apparently small minority of cases with sensory involvement would be wrongly excluded, but that would still be better than excluding virtually all global south cases as must currently be the norm.

## Data Availability

All clinical data relating to the cases described is included in the manuscript text.
